# Toxicology of Blister Agents: Is Melatonin a Potential Therapeutic Option?

**DOI:** 10.3390/diseases9020027

**Published:** 2021-04-10

**Authors:** Alejandro Romero, Eva Ramos, Francisco López-Muñoz, Cristóbal De Los Ríos, Javier Egea, Emilio Gil-Martín, René Pita, Juan J. Torrado, Dolores R. Serrano, Antonio Juberias

**Affiliations:** 1 Department of Pharmacology and Toxicology, Faculty of Veterinary Medicine, Complutense University of Madrid, 28040 Madrid, Spain; eva.ramos@ucm.es; 2 Faculty of Health Sciences, University Camilo José Cela, C/Castillo de Alarcón 49, Villanueva de la Cañada, 28692 Madrid, Spain; flopez@ucjc.edu; 3 Neuropsychopharmacology Unit, Hospital 12 de Octubre Research Institute (i+12), Avda. Córdoba, s/n, 28041 Madrid, Spain; 4 Portucalense Institute of Neuropsychology and Cognitive and Behavioural Neurosciences (INPP), Portucalense University, R. Dr. António Bernardino de Almeida 541, 4200-072 Porto, Portugal; 5 Thematic Network for Cooperative Health Research (RETICS), Addictive Disorders Network, Health Institute Carlos III, MICINN and FEDER, 28029 Madrid, Spain; 6 Health Research Institute, Hospital Universitario de la Princesa, 28006 Madrid, Spain; cristobal.delosrios@inv.uam.es (C.D.L.R.); javier.egea@inv.uam.es (J.E.); 7 Molecular Neuroinflammation and Neuronal Plasticity Research Laboratory, Hospital Universitario Santa Cristina, Instituto de Investigación Sanitaria-Hospital Universitario de la Princesa, 28006 Madrid, Spain; 8 AgroBioTech for Health ABH1 Group, Department of Biochemistry, Genetics and Immunology, Faculty of Biology, University of Vigo, 36310 Vigo, Spain; egil@uvigo.es; 9 Chemical Defense Department, Chemical, Biological, Radiological, and Nuclear Defense School, Hoyo de Manzanares, 28240 Madrid, Spain; 10 Department of Pharmaceutics and Food Technology, School of Pharmacy, Complutense University of Madrid, 28040 Madrid, Spain; torrado1@farm.ucm.es (J.J.T.); drserran@ucm.es (D.R.S.); 11 Centro Militar de Farmacia de la Defensa (CEMILFARDEF), Base Logística de San Pedro, Colmenar Viejo, 28770 Madrid, Spain; ajubsan@oc.mde.es

**Keywords:** sulfur and nitrogen mustard, melatonin, oxidative stress, inflammation, DNA damage, safety, galenic formulation

## Abstract

Blister or vesicant chemical warfare agents (CWAs) have been widely used in different military conflicts, including World War I and the Iran-Iraq War. However, their mechanism of action is not fully understood. Sulfur and nitrogen mustard exert toxic effects not only through the alkylation of thiol-bearing macromolecules, such as DNA and proteins, but also produce free radicals that can develop direct toxic effects in target organs such as the eyes, skin, and respiratory system. The lack of effective treatments against vesicant CWAs-induced injury makes us consider, in this complex scenario, the use and development of melatonin-based therapeutic strategies. This multifunctional indoleamine could facilitate neutralization of the oxidative stress, modulate the inflammatory response, and prevent the DNA damage, as well as the long-term health consequences mediated by vesicant CWAs-induced epigenetic mechanisms. In this context, it would be essential to develop new galenic formulations for the use of orally and/or topically applied melatonin for the prophylaxis against vesicant CWAs, as well as the development of post-exposure treatments in the near future.

## 1. Introduction

**Figure 1:**
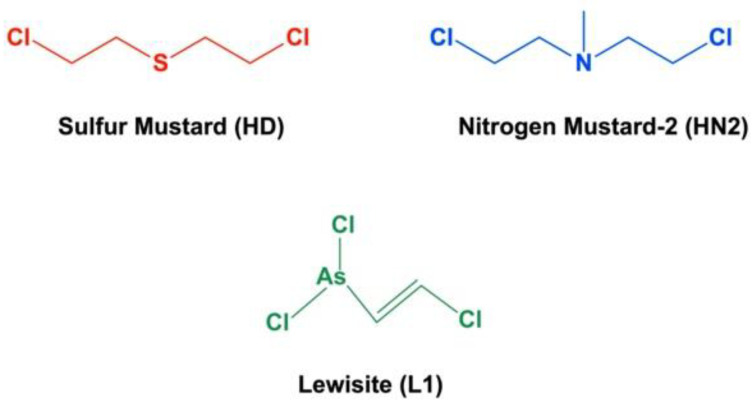
Chemical structure of the most representative blister agents.

Vesicants (blistering agents) are chemical warfare agents (CWAs) that cause blistering lesions in the skin and mucous membranes. This group of CWAs includes sulfur mustards (NATO code H, HS or HD), nitrogen mustards (HN), and lewisite (L) (Figure 1). Sulfur mustard (SM), bis(2-chloroethyl)sulfide, is the main blister agent and was introduced as a CWA during World War I. It was also widely used in the 1980s during the Iran-Iraq War and, more recently, DAESH terrorist attacks with crude SM have taken place in Iraq and Syria [1]. Nitrogen mustards and derivatives such as melphalan, chlorambucil, and cyclophosphamide are also alkylating agents used as cancer therapeutic agents [2]. Nonetheless, these cytotoxic alkylating agents were initially developed as chemical weapons used to induce ocular, dermal, and respiratory damage that results in immediate casualties, reduction in fighting efficiency, and demoralization. Thereby, adverse effects are of main concern. Indeed, there is a lack of knowledge concerning the complex mode of action of blister agents, and it continues to be a research focal point. Possible long-term effects after poisoning are up to now unknown.

Sulfur and nitrogen mustards undergo intramolecular cyclization reactions, giving rise to electrophile sulfonium or immonium ions, respectively [3]. These intramolecular reactions are favored by the presence of water and high temperatures [4]. For this reason, moist body areas are the most susceptible. Sulfonium ions are potent alkylating agents of biological molecules such as DNA, RNA, and proteins such as glutathione (GSH).

Unfortunately, total immediate decontamination after vesicant CWAs exposure is difficult to achieve nowadays. There are no completely effective antidotes nor effective treatments. In this context, melatonin, an excellent and broad-spectrum antioxidant agent, widely distributed in the body at appropriate concentrations [5], easily transported across morphophysiological barriers [6], and endowed with very low toxicity [7], has been proposed as a feasible agent to counteract induced toxic damage and possible long-term effects of the most representative blister agents. It is a well-characterized antioxidant; its anti-inflammatory and scavenger action has been widely described [8,9,10]. Specifically, in several tissues, such as the central nervous system (CNS) [11], skin [12], lung [13], and eyes [14] among others, and in presence of toxic agents such as metals [15], herbicides [16], cytotoxics [17], ethanol [18], etc. At this point, it arises as a promising protective agent against vesicants.

## 2. Target Organs and Acute Toxicity of Blister Agents: Focus on Melatonin Therapy and Safety Profile

The information available on the clinical effects of vesicants pertains mainly to SM use during World War I and the Iran-Iraq War [19].

After exposure to SM, there is an asymptomatic latency period before the first clinical signs and symptoms appear. This latency period varies between 2 and 48 h depending on the dose, temperature, humidity, and area of the body exposed [20]. The most sensitive areas are the thinnest and the most humid: the respiratory tract and eyes, and, on the skin, the axillae, neck, elbow creases, groin, genitals, and perineum. Ingestion of the agent can also cause direct gastrointestinal lesions [21].

**Figure 2:**
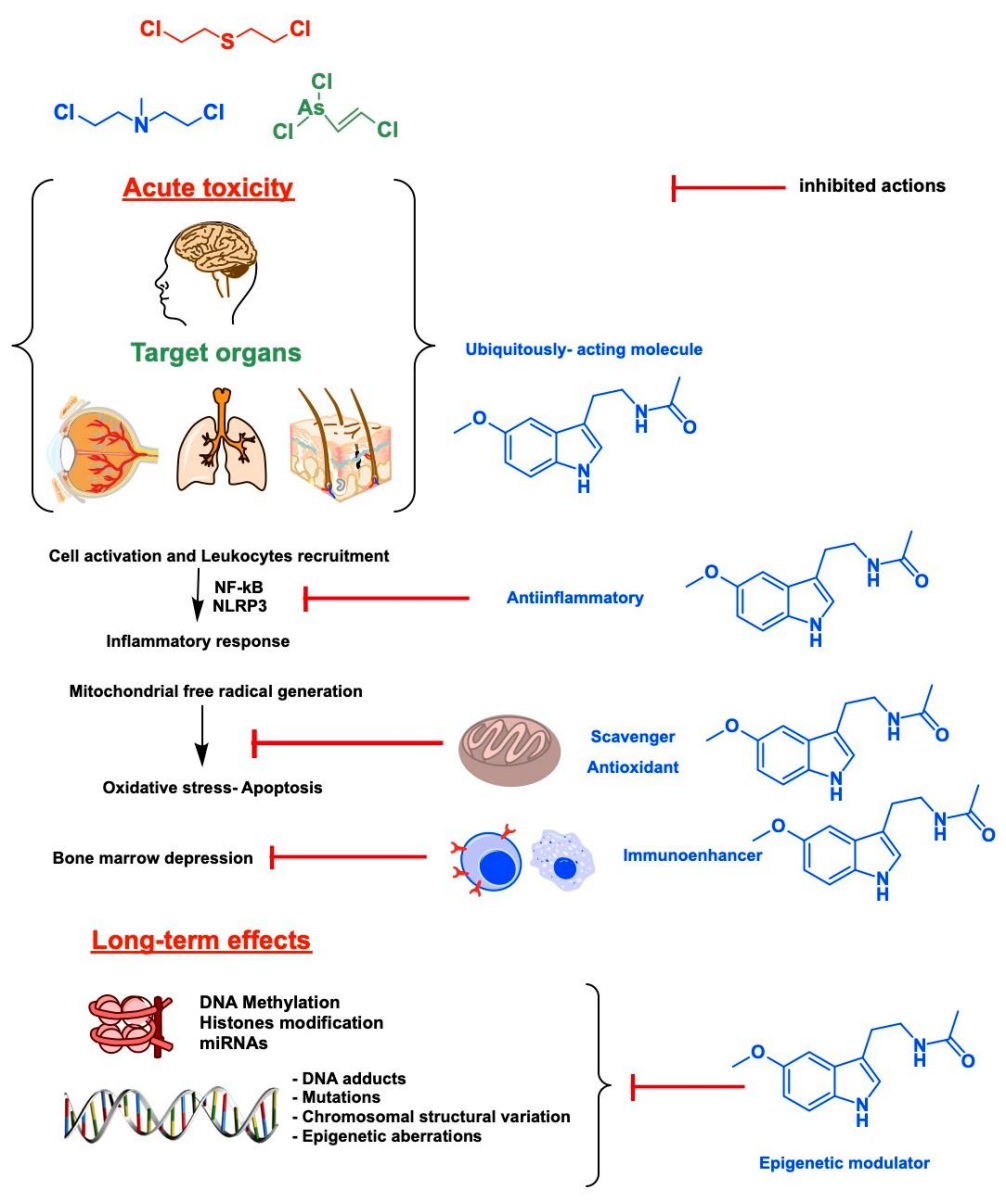
Summary of the cellular and molecular mechanisms displayed by melatonin against vesicant chemical warfare agents (CWAs).

Skin is susceptible to mustards-induced damage because is the main tissue in contact with mustards during exposure [22]. Mustards quickly absorb and penetrates the skin to initiate oxidative processes with high levels of ROS generation and accumulation, which induce damage of lipids, proteins, DNA, as well as deplete the non-enzymatic and enzymatic antioxidant defense systems of the skin. Erythema appears after the latency period and, subsequently, blisters develop on the erythematous areas. In severe lesions, particularly those due to exposure to the liquid agent, a large amount of exudate occurs, leading to infections by different microbial agents and necrosis [23]. Reepithelization is slow due to DNA alkylation, which prevents the epidermal cells from proliferating at a normal rate. Conversely, reepithelization of lesions caused by lewisite is faster than in the case of mustards. In this regard, direct contact with lewisite causes immediate irritation and pain at the site of contact. However, an effective antidote is not available and the current management against mustards is symptomatic and supportive [24]. In this context, a large amount of evidence supports the important role of melatonin in the human skin [12,25,26,27,28], where this indoleamine is synthesized at high concentrations for its local cytoprotective action [29]. Interestingly, in the human epidermis, melatonin displays excellent capacities against the damage caused by blister agents: (i) It can be metabolized to Cyclic 3-hydroxymelatonin (c3OHM), N^1^-acetyl-N^2^-formyl-5-methoxykynuramine (AFMK), and N^1^-acetyl-5-methoxykynuramine (AMK), which are metabolites highly effective in scavenging the free radicals produced by mustard exposure [30]. (ii) Melatonin modulates the nuclear factor erythroid 2–related factor 2 (Nrf2), the master regulator of antioxidant and cellular protective genes that counteract oxidative stress. It stimulates and preserves the major antioxidative enzymes, among them the GSH, reducing the concentration of free radicals and preserving the integrity and function of the cell membrane [31] (Figure 2). (iii) Melatonin contributes to reducing the inflammatory response and DNA damage by inhibiting the proinflammatory transcription factor NF-κB [32] and NLRP3 pathways [33,34] (Figure 2). (iv) Bacterial skin infections by mustard exposure may be prevented by the antimicrobial effect of melatonin [35]. Therefore, after mustard exposure, the melatonin supplementation may not only reduce the oxidative damage and alkylation of cellular macromolecules [36] but also improve the cutaneous regenerative potential [37].

Acute poisoning by SM is characterized by hoarseness and productive cough. In severe cases, noncardiogenic pulmonary edema may develop. Lesions may give rise to pseudomembranes between proximal and distal parts of the airways, resulting in obstruction [38]. Death in the first 24 h after exposure is usually due to acute respiratory failure, as a result of the bronchial tree obstruction by these pseudomembranes and laryngospasm. However, death after the first 3 days is usually due to bacterial pneumonia [39]. In this regard, melatonin has been shown to protect mustard-induced lung toxicity [40,41] or in combination with other drugs [42]. On the other hand, there is no direct information on the effects of lewisite on the respiratory tract in humans, but severe irritation may arise upon contact with the vapor. Pulmonary capillaries seem to be more susceptible to the action of lewisite [43].

Eyes are highly sensitive to mustards and the latency period for the onset of ocular signs and symptoms is shorter than for the skin symptoms [44]. Initial symptomatology includes intense irritation and temporary blindness due to palpebral edema. Ocular irritation progresses to conjunctivitis with photophobia, blepharospasm, pain, and corneal lesions in severe cases. In this context, the pleiotropy of melatonin has also been tested in several ocular pathologies. Thus, it has been recently reported its capacity to protects corneal epithelial cells from oxidative damage and reduce inflammation in dry eye disease (DED) [45]. It reduces cataract formation in rats [46] and can neutralize several etiopathogenic mechanisms in glaucoma [47], diabetic retinopathy [48], or macular degeneration [14]. Furthermore, in combination with selenium melatonin was able to decrease the oxidative damage in patients with ocular ischemic syndrome [49]. Therefore, as a single agent or in combination with other drugs, melatonin may be an excellent pharmacological option to counteract the mustards-related ocular diseases.

As above mentioned, SM is absorbed through the skin and eyes, when inhaled, and even when ingested into the gastrointestinal tract. Once absorbed, the systemic effects occur mainly in bone marrow [50], the gastrointestinal tract [21], and the CNS [51]. The main systemic effect of mustards is generalized myelosuppression, so its action is often referred to as radiomimetic. Granulocytes and megakaryocytes seem to be the most susceptible to the action of SM. In the first 3 days, leukocytosis may occur, followed by leukocytopenia at 7 to 10 days after exposure. The development of severe leukocytopenia or aplastic anemia are predictors of poor clinical outcomes [50]. In this respect, melatonin is produced by immune system cells; therefore, it would be able to modulate immune functions and bone marrow depression [52] (Figure 2).

Nitrogen mustards have similar effects to sulfur mustards, but their effects on the CNS are more severe, probably involving the DNA damage mechanism [53]. In this complex scenario, the multiplicity of actions of melatonin in CNS modulating the immune response and neuroinflammation, as well as reducing the excitotoxicity through a plethora of biochemical cascades [54], make this indoleamine a promising agent against mustard-induced toxicity (Figure 2).

The most accepted role of melatonin is its involvement in the regulation of the sleep-wake-up cycle. Interestingly, the idea of supplementing with melatonin the advanced aged population to protect brain health arises after the discovery of its antioxidant properties and it has been extensively examined [55]. It has been proposed that endogenous melatonin is a potential key factor in promoting human health since the last century [55]. Nonetheless, new roles and actions of this neurohormone are currently being described [56,57,58,59]. In the latest decades, melatonin has elicited pleiotropic promising results preclinically protecting and regulating many aspects due to its safety profile and inexpensive cost. Accordingly, researchers are widely studying melatonin’s actions in humans in several pathologies [60,61,62,63].

Published reports strongly suggest that there is a lack of serious adverse effects after its supplementation [7]. Despite there are no serious adverse effects described, a particular population has been suggested to be monitored to clarify this relatively safe profile [7]. It is worthwhile mentioning that high doses (up to 100 mg) of melatonin, intravenously administered in healthy volunteers did not produce any adverse effects, and did not induce sedation [64].

In this respect, in the European Union (UE), registered preparations of melatonin had been authorized for treating insomnia from 2 mg and up. Lower doses are considered as dietary supplements, like in the US and Canada at a range of doses up to 10 mg. It is worthy to notice that in the technical data of UE-authorized preparations of melatonin, no severe adverse effects were observed up to 300 mg. Moreover, the placebo group of the Circadin study reported nearly an equal incidence of adverse effects as the treated group (EPAR for Circadin. Procedure No. EMEA/H/C/695).

It has been pointed out previously that allometric dose translations from animals to human equivalent dose (HED) for clinical trials design need to be accurate [65]. Considering reported data to date, it is really necessary to select the correct dose to reach a reliable therapeutic approach since it has been shown that incorrect dose translation seems to be the cause of a lack of success [66]. In this regard, It has been proposed an extrapolation of animal effective doses to humans using the body surface area (BSA) normalization method [65]. There is a discrepancy between the protective profile of melatonin in animals and humans. It seems to be due to a low dosage in humans compared to those administered in animals as a result of the HED [66,67].

Taken as a whole, using higher doses of melatonin in severe situations seems to be a good idea due to the lack of adverse effects and the numerous beneficial effects observed in animals at the HED. Nevertheless, developing more clinical trials with melatonin at high doses remains an ongoing challenge for the scientific community since it is an inexpensive nonpatentable molecule with limited interest for the pharmaceutical industry.

## 3. Epigenetics and Long-Term Toxicity of Vesicants: A New Niche for Melatonin That Deserves Attention

Apart from the direct blistering disease, large or prolonged exposure to vesicants originates dermal, ocular, and respiratory long-term harmful sequelae [68,69,70] that are currently devoid of pathogenic model [44,71]. Trying to find the molecular mechanism of these delayed health disablements, recent research is interrogating the involvement of epigenome [40,72,73], parallel to the growing importance that epigenetic modulation is acquiring in the etiopathogenesis of multiple disorders [74]. Regarding this, genotoxicity linked to SM such as detrimental inflammation or cardiovascular, respiratory, and neoplastic disease has been recently addressed from the perspective of their epigenetic plausibility [75,76,77]. Some observations support this assumption, such as the interleukin-dependent inflammation in normal human epidermal keratinocytes exposed to sulfur mustard, in which the release of pro-inflammatory mediators was preceded by the induction of targets from the p38-MAPK pathway [78], as well as the suppression, presumably dependent of histone deacetylase upregulation of antioxidant and anti-inflammatory genes in the “mustard lung” [73]. Definitely illuminating has been the report of an array of more than 50 antioxidant and oxidative stress-related genes with their pattern of expression modified in the lung tissue of SM victims [79].

Despite being under construction, the complex world of epigenetics has already demolished some clichés of 20th-century genetics. It is therefore conceivable that a consensus on triggering factors, maintenance, and transmission of epigenetic marks, and the spectrum of functions on which they can have effects, has not been reached yet. Moreover, the intricate epigenetic machinery includes a huge heterogeneity of mechanisms and modulators closely imbricated in the integrative control of gene expression. However, classically the three most prominent typologies for which a better molecular understanding and range of effects have been reported are DNA promoter methylation, chemical modification of chromatin, and non-coding microRNAs (miRNAs). We will refer to them in the following paragraphs because they have been also the modalities scrutinized, although very sporadically, for the clarification of the long-term damage caused by blistering agents.

Regarding methylation of CpG islands on gene promoters, compelling evidence has been provided that acute SM produces an extensive dose-dependent modulation on the methylation status of murine dermal cells [80]. Specifically, sub-lethal doses downregulated through hyper-methylation up to 37% of the 78 epigenetic loci screened. The relevance of these effects was revealed in the global DNA hyper-methylation detected in skin samples from an individual accidentally exposed to a few drops of sulfur mustard 1 year earlier [80].

Under the umbrella of “histone code” a wide group of post-translational modifications (acetylation, methylation, phosphorylation, etc.) critical for nucleosome structure is encompassed. The dynamical regulation of these chemical tags governs the accessibility of transcription machinery to the chromatin, which oscillates between an open active structure (euchromatin) amenable to transcription and the compact inactive heterochromatin unable to be transcribed [81]. Similar to DNA methylation, histone modifications are shedding light on genome functionality. Hence, the histological lesions produced in rat lungs after acute injection with the nitrogen mustard-derivative mechlorethamine (MEC) were significantly prevented by coadministration of the histone deacetylase inhibitor Trichostatin A and, in accordance with the above results, increased by MEC combined with decitabine, a DNA methyltransferase inhibitor [73].

On the side of miRNAs, evidence of the post-transcriptional regulatory intervention of some of them in the context of mustard intoxication has been recently collected in vitro from both keratinocytes [82,83,84] and early endothelial cells [85]. It is worthy of special emphasis in this regard the screening of urinary excretion of miRNA-9 and miRNA-143 in a cohort of 32 SM-exposed patients and 32 healthy individuals, which decreased in correlation with symptomatology [86].

Thanks to the visionary hypothesis of Korkmaz and Reiter [87] and ulterior experimental research [88], there is currently little doubt that deleting and reprint of epigenetic marks are part of melatonin pleiotropy. It has been established, for example, that epigenetic reprogramming may include the melatonin receptors, as reported for melatonin receptor 1 (MT1) upregulation in rat glioma cells treated with valproate, which involved histone acetylation [89]. Likewise, the oocyte nuclear receptors, whose activation by the hormone induces epigenetic modifications in the DNA superstructure that help transmit adaptive responses to the next generation [90]. Additionally, melatonin per se can induce long-term epigenetic changes in gene expression, as observed in litters of pregnant rats treated with the hormone that activated a genetic program of more than 400 kidney genes to prevent programmed hypertension in the adult offspring [91]. Of special mention is the benefit that melatonin provides to sleep disturbances commonly afflicting mustard-injured victims. The sleep quality of 30 male veterans from the Iran-Iraq War showing sleeplessness along with mild/moderate respiratory disease and nocturnal depletion of circulant melatonin, improved after supplementation with the indoleamine [92,93]. In this regard and analogously to shift workers, among which insufficient sleep reduces DNA methylation and the genetic risk of job-exhaustion is linked to a downstream variant of MT1 [94,95], back to healthy sleep in vesicant cytotoxic-affected victims may be linked with the epigenetic restoration of the epigenome by the exogenous melatonin.

Despite this background, the investigation undertaken to address the epigenetic potential of melatonin and its applicability to mucosal blistering and delayed manifestations of vesicant exposition is insufficient. However, direct and indirect evidence points out that melatonin has excellent abilities to reverse the epigenetic perturbations associated with vesicant poisoning [76], and therefore, to alleviate disturbances appearing long after exposure. Particularly remarkable in this sense is the improvement that melatonin provides to chronic obstructive pulmonary disease, closely related to mustard lung phenotype [96], which allowed contemplation of the therapeutic options of the hormone to manage the respiratory complications associated with high doses or prolonged mustard intoxication [72,75].

In sum, the elucidation of epigenetic disturbances that blistering agents cause is a step forward in bridging new approaches and therapeutic targets to remedy their long-term insidious disease. For this challenge, the multitasking potential of melatonin, including the ability to manipulate gene editing and reprogramming, must be considered [97,98,99]. Thus, the epigenetic modifications induced by vesicants and their eventual reversion by melatonin deserve attention for reaching new strategies of supportive treatment. Specifically, more in vitro research in addition to experimentation with animal models and systemic controlled clinical trials must be carried out. The goal is relevant because melatonin-based palliative countermeasures to treat late complications against future accidents or war conflicts [100] may be in our hands in the short term.

## 4. Reaction Mechanisms of Melatonin with Blistering Agents (HAT Mechanism and Counteract DNA Damage)

**Scheme 1:**
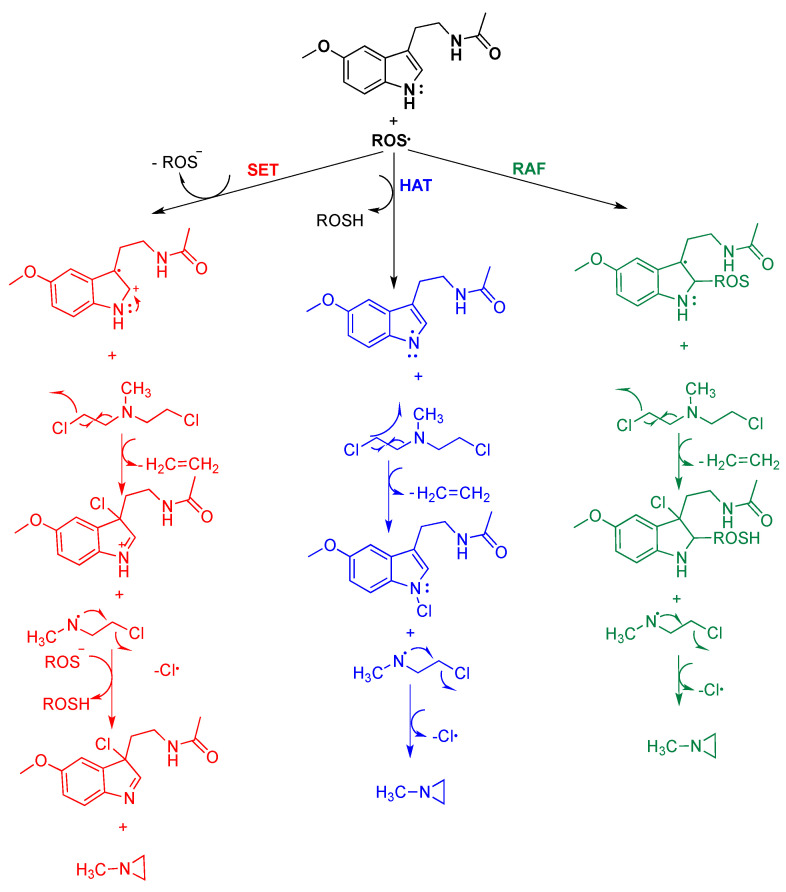
Three alternative free radical-mediated mechanisms for quenching the nitrogen mustard toxic agent exerted by melatonin.

Previously, our research group reported through computational calculations the hydrogen-atom transfer (HAT) as the suitable mechanism to quench SM, furnishing the much less toxic thiirane [36,101]. The recognized free radical scavenging capacity of melatonin also facilitates the neutralization of mechlorethamine-induced toxic damage. In this context, three mechanisms could also be proposed to achieve this action (Scheme 1).

The widely extended HAT mechanism would quench mechlorethamine (HN2) to form the much less toxic *N*-methylaziridine. In this regard, melatonin would reduce reactive oxygen species (ROS) generation mostly by transferring the hydrogen at N1. Afterward, radical melatonin attacks mechlorethamine by radical nucleophilic substitution, and the reactive intermediate collapses by releasing ethylene and subsequently a chlorine atom, thereby forming *N*-methylaziridine. Otherwise, single electron transfer (SET) and radical adduct formation (RAF) are alternative mechanisms that produce the same by-product when neutralizing mechlorethamine. In these last mechanisms, the pyrrole double bond quenches ROS either forming an adduct (RAF mechanism) or transferring an electron (SET mechanism). In both cases, the reactive melatonin derivative intermediates neutralize mechlorethamine, similar to HAT. Indeed, the analysis of the final melatonin derivatives formed could clarify which of these mechanisms is more plausible.

Immonium cations are highly electrophilic and can alkylate nucleophilic molecules such as those enzymes containing thiol groups, which are responsible for regulating Ca^+2^ homeostasis within the cell. Such processes would increase the intracellular Ca^+2^ concentration, disrupting the microfilaments responsible for cell integrity, with the subsequent activation of endonucleases, proteases, and phospholipases that finally induce apoptosis. Moreover, mustards interact with GSH and increase the concentration of free radicals that, through peroxidation of membrane lipids, affects the integrity and function of the membrane.

**Scheme 2:**
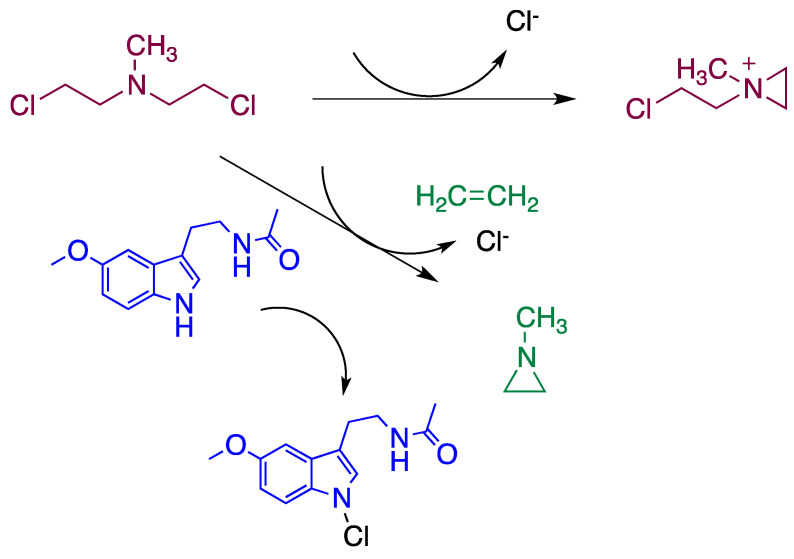
First cyclization reaction that forms the highly electrophilic ammonium cation. Melatonin prevents this cation by alternatively forming the less toxic *N*-methylaziridine (a HAT-type mechanism is represented as an example).

DNA alkylation produces cross-linking and breakage of the strand; and polymerases such as poly (adenosine-ribose diphosphate) polymerase (PARP) are activated leading to depletion of the substrate nicotinamide adenine dinucleotide (NAD^+^), inhibition of adenosine triphosphate (ATP) synthesis, and finally apoptosis. Therefore, the fastest-dividing cells are the most affected targets of mustards. On the other hand, mechlorethamine appears to have a specific affinity for nitrogen at position seven of guanine. As anticipated above, melatonin or its reactive species would transform mechlorethamine in the much less toxic *N*-methylaziridine (Scheme 2).

**Scheme 3:**
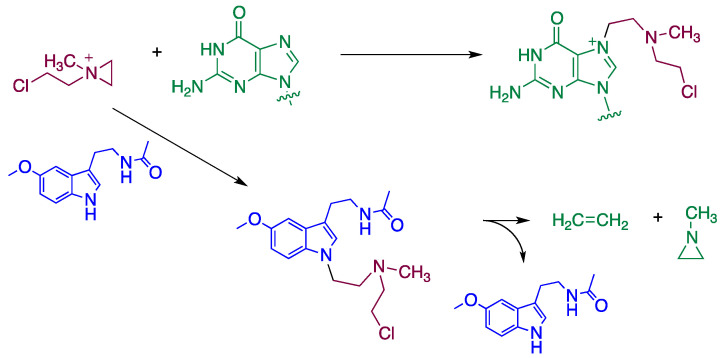
Formation of reactive nitrogen mustard and interaction with guanine in DNA. Melatonin would be able to trap the ammonium cation, avoiding such adduct formation.

**Scheme 4:**
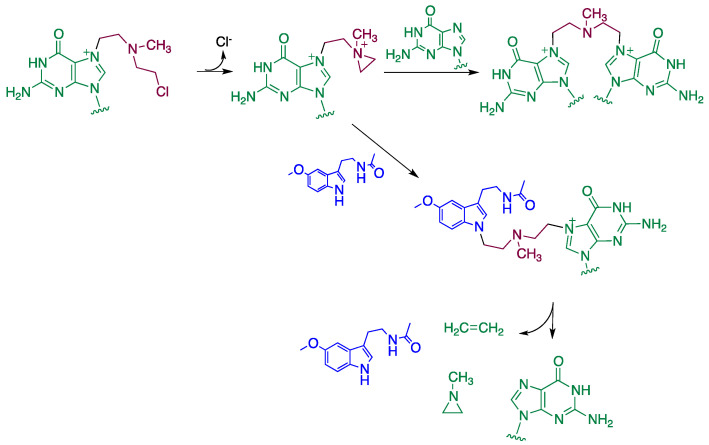
Second cyclization and alkylation of complementary DNA base resulting in a DNA crosslink.

Scheme 2 shows how melatonin could dissipate the generation of the highly electrophilic ammonium cation. Otherwise, this reactive species is thought to form an adduct with guanidine at N3 (Scheme 3). In this scenario, melatonin could sequester the immonium cation, turning it into *N*-methylaziridine, and thus, preventing the formation of the guanidine-nitrogen mechlorethamine adduct (Scheme 4).

It is worthwhile mentioning that the toxicity mechanism of the mechlorethamine guanidine adduct comprises DNA-cross-linking with another guanidine base (Scheme 3), through the formation of an intermediate adduct that presents the corresponding cyclic ammonium cation structure. In this situation, melatonin likely counteracts cross-linking by reacting again with the ammonium cation, herein bond to a guanidine. If that happens, melatonin comprises the potential to catch the mechlorethamine bridge, thereby removing it from the guanidine.

## 5. New Melatonin Galenic Developments Addressed to Reduce the Toxicity of Blister Agents

Most melatonin marketed products are formulated as oral food supplements or drugs up to 5 mg [102]. Melatonin not only regulates the sleep-wake cycle, but it is also a natural antioxidant and anti-inflammatory substance with interesting characteristics to treat blister agent toxicity. However, doses higher than 5 mg are required to achieve these effects. Additionally, melatonin action must last for hours. Therefore, the conventional immediate oral formulations must be replaced by sustained-release oral products. It is also interesting to develop topical and parenteral melatonin formulations to treat cutaneous, ocular, and acute effects.

To avoid melatonin degradation, as well as improve the antioxidant activity of melatonin, other antioxidant agents such as vitamin E can be incorporated into the formulations [103]. In this regard, the oxidation of melatonin has been previously described by Reiter et al. [104].

### 5.1. Oral Formulations

According to the biopharmaceutical classification system, melatonin is a type II drug with low solubility and high permeability. Melatonin dissolution rate can be improved by the association to silica dioxide, to increase its specific surface [105]. Moreover, in these formulations, the addition of HPMC phthalate (HP55) seems helpful to extend drug release and to improve bioavailability between 2 and 4 times. Silica dioxide can be replaced by starch to achieve similar effects [106]. It has also been reported in fast dissolving tablets with doses between 3 and 60 mg of melatonin for pediatric application. Suitable excipients are mannitol, crospolyvinylpyrrolidone, lactose, magnesium stearate, silica dioxide, tartaric acid, and sodium bicarbonate [107]. Additionally, Lee et al. [108] have prepared matrix tablets based on high viscosity HPMC (100,000 cps) to obtain sustained release for up to 8 h. Moreover, 20 mg melatonin tablets can be obtained by direct tableting based on the following composition: HPMC (92.4%), melatonin (1.6%), microcrystalline cellulose (4%), magnesium stearate (1%), and silica dioxide (1%). Working with suitable excipients such as lactose, Gelucire^®^ 50/13, stearic acid, carnauba wax, silica dioxide, and alginic acid, Albertini and colleagues [109] suggested using lipid excipients to delay melatonin release rate in pediatric formulations.

### 5.2. Topical Formulations

Escames and Acuña [110] reported a topical composition of melatonin between 2.5 and 5% for the prevention and treatment of mucositis of the digestive tract. The suitable topic vehicle was gel-based on Pluronic F127^®^ mixed with preservatives. The suggested oral dose was 15 mg 3 times per day. In this context, the addition of Q10 at 0.2 and 1.5% can increase its bioavailability [111].

Kirkawai et al. [112] studied different possible bioavailability enhancers. Generally, the higher the solubility in the vehicle the lower the enhancement permeation effect. Best results were obtained with isopropyl myristate, lauroglycol FCC, and ethanol. A lower permeability effect was observed with Labrasol^®^, propylene glycol, and mineral oil. In this regard, Oh et al. [113] described oleic acid as the best topic permeability enhancer for melatonin formulations. Diethylene glycol monoethyl ether has also been proposed as a permeability enhancer of melatonin [114].

Furthermore, nanoencapsulation was suggested by Hoffmeister et al. [115] to prolong topical release and activity of melatonin formulations. Similar effects have been obtained with transdermal patch technology with limonene as a permeability enhancer of melatonin [116].

### 5.3. Parenteral Formulations

The low solubility of melatonin is the main concern to formulate parenteral products. The water solubility of melatonin at 25 °C is approximately 1.8 mg/mL [117]. A half-life of 33 days and poor chemical stability especially after exposition to light has also been reported for melatonin [118,119]. Complexation with polyvinylpyrrolidone K12 and freeze-drying increase melatonin´s water solubility up to 9.9 mg/mL [117]. A combination of sodium dodecyl sulfate and dodecyl sulfosuccinate at a 2:1 ratio was useful to increase melatonin solubility [120]. It has also been suggested to use lipid excipients to prepare liposomes and nanoparticles with a moderate solubility of around 3 mg/mL and prolonged effects [121].

Different cyclodextrins have been also proposed to improve solubility and permeability. For instance, beta-cyclodextrin [122], hydroxypropyl beta-cyclodextrin and methylated beta-cyclodextrin [123,124].

In summary, melatonin is considered a very interesting active product useful for many potential applications. Depending on the administration route and the duration of effects, several formulations have been developed and reported.

## 6. Conclusions

There is plenty of literature related to the beneficial actions of melatonin and its application in numerous diseases. Melatonin displays a significantly high antioxidative capacity, anti-inflammatory actions and recent studies have shown its ability as a promising epigenetic modulator. We believe that the therapeutic value of melatonin represents an excellent opportunity for the treatment against vesicant CWAs. In this regard, the future research lines to incorporate the melatonin into vesicant poisoning treatment should be addressed to (i) establish what melatonin doses are capable of counteracting blistering-related toxicity; (ii) develop new prolonged-release preparations, to modify its pharmacokinetics, to achieve the prophylaxis and post-exposure treatments against vesicant CWAs; (iii) corroborate the high safety profile of melatonin, another pivotal reason for further research on the field of vesicant CWAs, especially since none of the current treatments act as effective antidote; (iv) screening melatonin as a prophylactic agent or in adjuvant therapy to test possible therapeutic synergies with drugs in use and/or the reduction of their side effects, which would provide interesting beneficial perspectives, and (v) according to this potential, elucidate the role that can play melatonin in the epigenotoxicity of vesicant CWAs and their long-term detrimental effects. Perhaps soon, the multiple benefits of melatonin supplementation to treat vesicants exposure and long-term sequelae will become a reality.

## Data Availability

Not applicable.
